# A Deep Multi-Task Learning Framework for Brain Tumor Segmentation

**DOI:** 10.3389/fonc.2021.690244

**Published:** 2021-06-04

**Authors:** He Huang, Guang Yang, Wenbo Zhang, Xiaomei Xu, Weiji Yang, Weiwei Jiang, Xiaobo Lai

**Affiliations:** ^1^ College of Medical Technology, Zhejiang Chinese Medical University, Hangzhou, China; ^2^ Cardiovascular Research Centre, Royal Brompton Hospital, London, United Kingdom; ^3^ National Heart and Lung Institute, Imperial College London, London, United Kingdom; ^4^ College of Life Science, Zhejiang Chinese Medical University, Hangzhou, China

**Keywords:** automatic segmentation, brain tumor, deep multi-task learning framework, multi-depth fusion module, magnetic resonance imaging

## Abstract

Glioma is the most common primary central nervous system tumor, accounting for about half of all intracranial primary tumors. As a non-invasive examination method, MRI has an extremely important guiding role in the clinical intervention of tumors. However, manually segmenting brain tumors from MRI requires a lot of time and energy for doctors, which affects the implementation of follow-up diagnosis and treatment plans. With the development of deep learning, medical image segmentation is gradually automated. However, brain tumors are easily confused with strokes and serious imbalances between classes make brain tumor segmentation one of the most difficult tasks in MRI segmentation. In order to solve these problems, we propose a deep multi-task learning framework and integrate a multi-depth fusion module in the framework to accurately segment brain tumors. In this framework, we have added a distance transform decoder based on the V-Net, which can make the segmentation contour generated by the mask decoder more accurate and reduce the generation of rough boundaries. In order to combine the different tasks of the two decoders, we weighted and added their corresponding loss functions, where the distance map prediction regularized the mask prediction. At the same time, the multi-depth fusion module in the encoder can enhance the ability of the network to extract features. The accuracy of the model will be evaluated online using the multispectral MRI records of the BraTS 2018, BraTS 2019, and BraTS 2020 datasets. This method obtains high-quality segmentation results, and the average Dice is as high as 78%. The experimental results show that this model has great potential in segmenting brain tumors automatically and accurately.

## Introduction

Glioma is the most common and aggressive brain tumor. It is diffuse and can spread to any part of the brain, which makes it difficult to detect (1). According to the statistics, the annual incidence of glioma is about 5 cases per 100,000 people (2, 3). Although gliomas are less common than other fatal diseases, they are poorly treated and have a higher mortality rate (4). The World Health Organization (WTO) divides gliomas into 4 grades, grades 1 and 2 are low-grade gliomas (LGG), and grades 3 and 4 are high-grade gliomas (HGG), which grade 4 glioma patient survival time of less than one year (1). Therefore, early diagnosis plays a vital role.

Magnetic resonance imaging (MRI) is an important method in medical imaging diagnosis (5). It can visualize interesting parts of the brain, generate multi-modal images, and provide more information of the tumor, so it is widely used in clinical diagnosis, treatment, and surgical planning (6). Generally, clinicians manually segment brain tumor regions on two-dimensional MRI slices or planar projections to facilitate the implementation of the next treatment plan (7). However, this method of segmentation is laborious and subjective (6). A patient usually has hundreds of slices, manual segmentation will consume a lot of time and energy for the doctor. Moreover, the boundaries of brain tumors are blurred and it is difficult to distinguish from healthy tissues, which also makes the segmentation results of experts differ (8, 9). Therefore, it is urgent to use computer technology to realize automatic segmentation of medical images. In recent years, with the development of deep learning, convolution neural networks (CNN) has been widely used in the field of medical image segmentation, and CNN has become the main method for its high efficiency and time-saving. In this paper, brain tumor segmentation is a multi-category segmentation task, which often requires a more complex network structure to obtain the ideal segmentation result. Based on this, we propose a new network structure to segment brain tumors, called the deep multi-task learning framework. The contributions of this article are mainly in the following aspects:

- In this paper, we propose a deep multi-task learning framework to improve the discontinuity problem of mask boundary prediction. It adds a distance transform decoder based on V-Net, which regularizes the mask decoder to ensure the smoothness of segmentation prediction.
- We also propose the use of linear weighting to combine the loss function of mask prediction and distance estimation tasks, and discuss the impact of weight changes on segmentation accuracy. Besides, we also set weights in the loss function of mask prediction to reduce the model’s attention to the background.
- We integrate the multi-depth fusion module into the down-sampling stage, which can effectively fuse global information and local information, improving the ability of the network to extract features. In the control group experiment, we remove the multi-depth fusion module and find that the accuracy was reduced, which can verify the effectiveness of the module in improving accuracy.

The rest of this article is as follows. Section 2 describes the development of image segmentation, related research on MRI segmentation of brain tumors, and a summary of the work of the article. In Section 3, we elaborated on the proposed method, including data preprocessing, the principle of the model, and the loss function. In the fourth section, we give the details of the experiment. Then we presented and discussed the experimental results in Section 5, and finally summarized the conclusions in Section 6.

## Related Works

### Traditional Image Segmentation Method

The earliest and most traditional image segmentation method is based on threshold segmentation. The basic principle of the threshold segmentation method is to divide the image pixels into the target area and the background area by setting the characteristic threshold. Taheri S. et al. used a threshold-based method to segment three-dimensional brain tumors, and adopted two threshold update schemes for searching and self-adaptation, achieving automatic or semi-automatic segmentation according to the complexity of the tumor shape (10). A more widely used algorithm is a segmentation algorithm based on edge detection, which is one of the most studied methods. Max W.K. et al. proposed an edge detection method based on weighted local variance for blood vessel boundary segmentation, which is robust to changes in edge intensity contrast (11). But the edge segmented by this method is not continuous.

### Machine Learning

The segmentation method of machine learning is mainly divided into two categories: supervised learning and unsupervised learning. Supervised learning includes KNN, Bayes, and ANN algorithms. Unsupervised learning mainly includes some clustering methods, such as K-means, FCM, etc. Anbeek P. et al. applied the KNN algorithm to skull MRI to segment multiple sclerosis lesions. This method uses voxel position and signal strength to determine the probability of each voxel lesion to generate probabilistic segmentation images (12). In order to improve the segmentation of brain PET images, Xia Y. et al. proposed to incorporate the *a priori* anatomical knowledge represented by the probabilistic brain atlas into the variational Bayes to segment gray matter, white matter, and cerebrospinal fluid in brain PET-CT images (13). Franklin S.W. et al. used ANN technology based on Gabor and moment-invariant features to segment the retinal blood vessels in the fundus to accurately obtain the width of the blood vessels (14). In medical image segmentation, the most commonly used clustering method is k-means clustering. Moftah H.M. et al. proposed an adaptive k-means clustering method, which maintains the best results in the iterative process and can effectively segment MR breast images (15). The fuzzy C-means algorithm based on objective function is also commonly used. Chen W. et al. applied the fuzzy c-means (FCM) clustering method to the wind field to segment breast lesions from MRI-enhanced images (16).

### Deep Learning

With the development of deep learning, the CNN is gradually applied to image segmentation, which greatly improves the accuracy of image segmentation. Fully Convolutional Networks (FCN) is the first network structure that successfully uses deep learning for image semantic segmentation. FCN converts the fully connected layers in classification networks such as AlexNet, VGG Net, and GoogLeNet into convolutional layers and applies them to segmentation tasks to achieve pixel-level semantic segmentation (17). Badrinarayanan V. et al. proposed a new semantic pixel segmentation network structure SegNet, which is based on the DeconvNet. Their innovation is to input feature maps during the up-sampling process to better restore the information lost during the down-sampling process (18). U-Net is a segmentation network with a simple structure proposed by Ronneberger et al., which is widely used in medical image segmentation because it can adapt to a small training set. It is similar to FCN, but the difference is that U-Net uses features to be stitched together in the channel dimension to form a thicker feature, while FCN adds features point by point (19). Although CNN is very popular, most methods can only process two-dimensional images, and most medical data used in clinical practice is composed of three-dimensional volumes. Milletari et al. proposed a three-dimensional image segmentation network based on U-Net. Their network is trained end-to-end on the 3D MRI of the prostate and can predict the segmentation of the entire 3D image at once (20). Liu et al. proposed a new convolutional neural network, which consists of three independent sub-networks, including an improved ResNet50, a feature pyramid attention network and a naive decoder network. The three networks are connected to form an end-to-end prostate segmentation model (21). Ding et al. proposed a fuzzy information deep learning segmentation (Fl-DL-Seq) network to segment infant brain tissue. They use the volumetric fuzzy pooling (VFP) layer to model the local fuzziness of the volume convolution map by fuzzing, accumulating and deblurring the neighborhood of the adjacent feature map (22).

### Brain Tumor Segmentation

Although a large number of neural network structures have achieved high segmentation performance in the segmentation field, they are not necessarily adaptable to the field of brain tumors. Due to the complexity of multi-modal brain tumors, in order to obtain clinical segmentation effects, the network structure must be designed according to the characteristics of brain tumor MRI images, for this reason, many experts have done a lot of research. Lachinov D. et al. proposed a deep cascade method for automatic brain tumor segmentation, which modified the three-dimensional U-Net architecture to effectively process multi-modal MRI input. They used multiple encoders to make each individual mode independently generating a corresponding feature map (7). Feng X. et al. proposed to use a set of three-dimensional U-Net with different hyperparameters to segment brain tumors. They trained the six networks with different encoder/decoder block numbers, different input patch sizes, and different loss weights, and finally performed integrated modeling (23). Lele C. et al. proposed a model based on 3D convolutional neural networks. Their innovation lay in extracting features from two different scales to obtain multi-scale context information. They also proposed a new structure, that was, according to the characteristics of the brain tumor lesion area, the lesion sub-regions were stratified (24). Zhou C.H. et al. proposed a lightweight deep model based on the model cascade (MC) strategy, a one-time multi-task network (OM-Net), which could better solve the problem of class imbalance. Besides, they also designed a cross-task guided attention (CGA) module that could adaptively recalibrate the channel characteristics (25). Myronenko A. et al. designed a variational autoencoder (VAE) branch to reconstruct the input image in a network based on the encoder-decoder architecture. The function of the VAE branch was to jointly reconstruct the input image and the segmented image to standardize the shared encoder (5). Zhang et al. proposed a brain tumor segmentation model based on multi-encoders. Each modal image corresponds to a down-sampling path. This one-to-one feature extraction method reduces the complexity of the feature extraction (26).

### Our Work

Segmentation of brain tumor MRI data based on multimodality is challenging for the following reasons. First of all, brain tumors may show similar characteristics with glial hyperplasia and stroke, which is easy to cause confusion (1). Second, brain tumors can appear in any part of the brain. Besides, the size, shape, and appearance of brain tumors in different patients are also different, which increases the difficulty of segmentation (27). Finally, the main difficulty of the brain tumor segmentation task is the imbalance between the classes. Since the lesion area is very small in most cases, the background area dominates, resulting in low segmentation accuracy (24). All these make the task of brain tumor segmentation more difficult.

**Figure 1:**
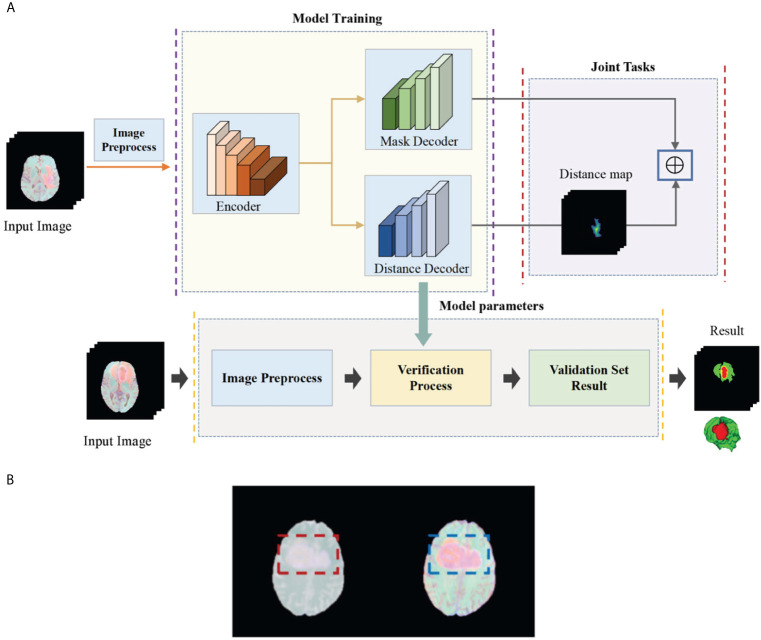
**(A)** Flow chart of our deep multi-task learning framework for brain tumor segmentation. **(B)** Brain tumor images before and after the standardization.

In this article, in order to deal with the above challenges, we propose a deep multi-task learning framework combined with a multi-depth fusion module. It is a derivative of the V-Net network structure. The traditional V-Net has only one decoder, which will produce discontinuous boundaries in the segmentation results. Therefore, we propose a parallel decoder architecture to perform distance estimation while predicting the mask to ensure the smoothness of the prediction result. As shown in **Figure 1A**, the first decoder is used to predict the mask, and the second decoder is used to estimate the distance map. The main function of the distance decoder is to regularize the mask prediction path to make the boundary of the mask smooth and continuous. In order to combine the two tasks, we propose a new loss function, which consists of two parts: the categorical focal loss of the mask decoder block and the mean square error of the distance transform decoder block. The final loss function is the weight of the above two sum up. We also set some weights for Categorical Focal loss to reduce the attention of the model to the background area and alleviate class imbalance. In order to improve the ability of the model to extract features, we integrate the multi-depth fusion module into the encoder (28). This module averages and fuses multi-level feature signals, which can effectively capture global features and local features. Based on the brain tumor data provided by the BraTS 2018, BraTS 2019, and BraTS 2020, we evaluate the model and compare it with the methods proposed by other researchers and participating teams. Experimental results show that this method has a good segmentation effect.

## Methods

The task of this paper is to segment brain tumors from three-dimensional MRI images. In order to obtain higher segmentation accuracy, we propose a deep multi-task learning framework based on V-Net and integrate the attention module in the encoder. **Figure 1A** shows the flow chart of all work. In this section, we will introduce in detail the preprocessing process and the structure of the deep multi-task learning framework.

### Pre-Processing Steps

The BraTS data all have four MRI sequences with different contrasts: fluid attenuation inversion recovery (FLAIR), T1-weighted (T1), T1-weighted contrast enhancement (T1-CE), and T2-weighted (T2), different contrast may lead to slow convergence or disappearance of gradients during training, so images must be standardized. We standardize the four modes separately and then merge the modes. Here we choose Z-score for standardization, that is, the image minus the mean divided by the standard deviation. In **Figure 1B**, we show the comparison images before and after standardization. The left side is before standardization and the right side is after standardization (**Figure 1B**). It can be seen that the characteristics of the tumor are more obvious after standardization. The standardized formula is as follows:

(1) $$\hat{X}=\frac{X-\bar{X}}{\sigma },$$

among them, $\hat{X}$ represents the normalized image, *X* represents the original image, $\bar{X}$ represents the mean value of the image, and *σ* represents the standard deviation of the image.

After standardization, the MRI images of four modal sequences with a size of 240×240×155×1 are combined to generate a three-dimensional image of four channels, and the combined image size is 240×240×155×4. Then split the Mask image, that is, each type of label image is used as a separate channel image. The original image size is 240×240×155×1, the generated size is 240×240×155×4, and then the one-hot operation is performed on each channel. The non-zero value in channel 0 is the background area, and the non-zero value in channel 1 is the gangrene area, the non-zero value in channel 2 is the edema area, and the non-zero value in channel 3 is the enhanced tumor area. Finally, a patch operation is performed on the image and the mask, and several images and masks with a size of 128×128×64×4 are generated.

### The Deep Multi-Task Learning Framework

**Figure 2:**
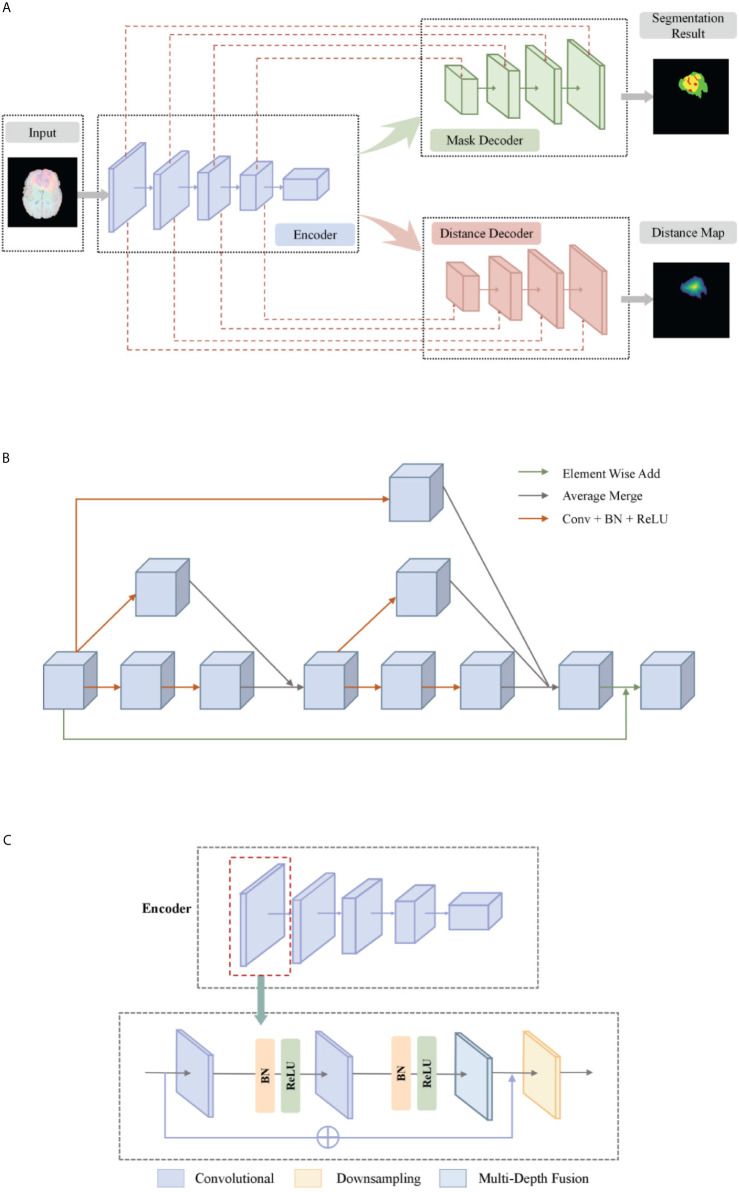
**(A)** The network structure of our deep multi-task learning framework. **(B)** Structure diagram of the multi-depth fusion module. **(C)** A detailed illustration of the encoder.

The basic idea of a deep multi-task learning framework is to implement feature information extraction at different resolution levels through alternately stacked convolutional layers and down-sampling layers. Then, the features extracted by the deconvolution joint encoder are used to realize the step-by-step resolution restoration and feature information restoration. Distance transform encoder helps smooth segmentation prediction and attention module helps feature extraction. Its network structure is shown in **Figure 2A**. We will elaborate on the innovation of the model from three aspects: the multi-depth fusion module, the distance transform decoder, and the loss function.

#### Multi-Depth Fusion

The multi-depth fusion module was originally used to perform whole-heart segmentation of CT images. Ye C. et al. applied the module to 3D U-Net to obtain the most advanced results (28). It is incorporated in the down-sampling part of our model. The structure of the multi-depth fusion block is shown in **Figure 2A**, and all convolution operations in the structure use a convolution kernel with a size of 3×3×3 and a stride of 1.

The operation of superimposing and averaging the signal that has undergone two convolution operations on the feature map and the signal that has undergone one convolution operation is called the fusion of feature maps of different depths. The input feature map is subjected to two different depth feature map fusion operations, and the input feature map is subjected to a convolution operation, and then the two signals are superimposed and averaged. Finally, the output signal is combined with the input feature map again as the final output of the module, its specific process is shown in **Figure 2B**.

This module averagely merges the characteristic signals of different depths, which can continuously merge local and global information. Compared with simply merging feature maps of different depths, the multi-depth fusion module adopts a better iterative layered fusion method. This architecture ensures that the deep feature map can effectively receive feature information from the shallow feature map. The final signal input and output resolution remain the same. The original encoder block consists of two to three convolutional layers and a downsampling layer. Now we place the multi-depth fusion module in front of each downsampling layer. The convolution still uses batch normalization and ReLU activation functions, just change the location of the ResNet connection. The specific structure is shown in **Figure 2C**.

#### Distance Transform Decoder

Our proposed network has two decoder modules with similar structures, and each decoder module is assigned a different task. The mask decoder module performs training mask segmentation according to pixel classification tasks, and the distance transform decoder module performs regression tasks to realize distance map estimation.

**Figure 3:**
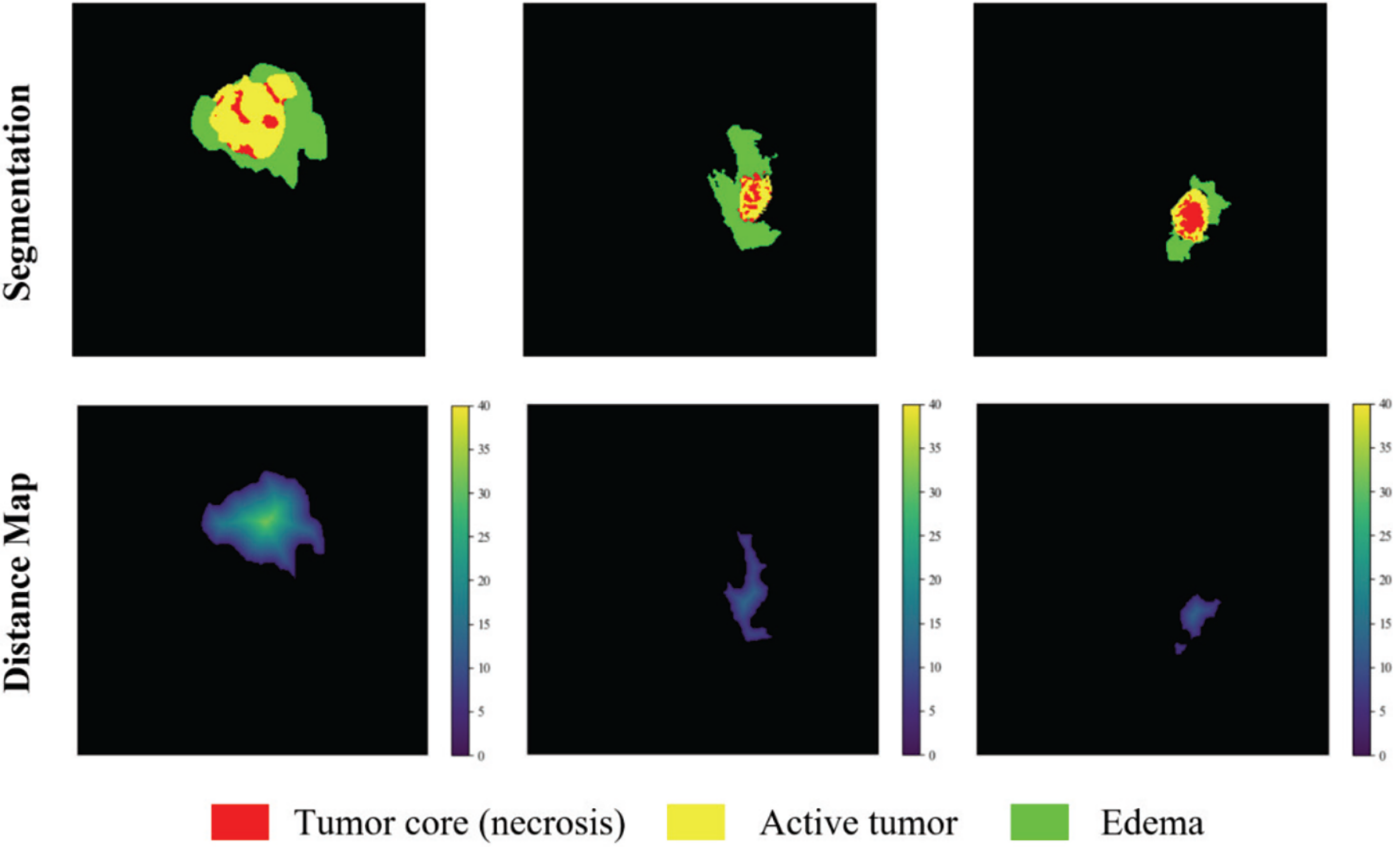
The example output results of the two decoders. Red color represents the tumor core (necrosis), yellow color represents the active tumor and green regions are the edema.

The distance transform decoder module is similar in structure to the mask decoder module. The image size is restored stage by stage by alternately stacking the deconvolution layer and the convolution layer, and the feature information extracted by the block-level connection joint encoder module is used to improve the predictive performance of brain tumor area contour. The difference with the mask decoder is the output channel in the distance transform decoder block is 3, which is equal to the number of input categories of the mask decoder block. We show in **Figure 3** an example of the mask decoder output and the distance decoder output comparison. In all the figures illustrate the segmentation results, red color represents the tumor core (necrosis), yellow color represents the active tumor and green regions are the edema.

#### Loss Function

The loss function of the model consists of two parts, the categorical focal loss of the mask decoder block and the mean square error of the distance transform decoder block. The final loss function learned by the optimizer is the weighted sum of the coefficients of the above two losses, where the distance graph prediction regularizes template prediction. The overall loss function formula is as follows:

(2) $$L_{\text{total}}=\lambda_{1}L_{\text{mask}}+\lambda_{2}L_{\text{dist}},$$

where *λ*_1_ and *λ*_2_ are the scaling factors, *L*_mask_ is the loss function of the mask decoder, and *L*_dist_ is the loss function of the distance transform decoder. The loss function of the mask decoder is shown in (3) and (4),

(3) $$L_{\text{FL}}=-y\text{log}y\prime \times {\left(1-y\prime \right)}^{\gamma }$$

(4) $$L_{\text{mask}}=L_{\text{FL}}\times W,$$

where *L*_FL_ is the pixel-by-pixel classification loss, *y* represents the true label, *y’* represents the predicted value after activation by Softmax, and *W* represents the category weight.

We choose categorical focal loss to solve the problem of imbalance between the foreground and background categories of brain tumor images. It adds a gamma factor to the two-class cross-entropy loss. Here we set the gamma factor to 2, so that the model reduces the loss of background voxels, and makes the model pay more attention to the target voxels that are difficult to segment and easy to error. Finally, in order to further adjust the category imbalance, we assign a specific weight to each type of label, the background label weight is assigned a value of 0.1, and the ET, WT, TC area label weight is assigned a value of 1.0. For the loss function of the distance transform encoder, we refer to the practice of Balamurali M. et al. (29), using the mean square error loss. The loss function of the distance transform decoder is shown in (5),

(5) $$L_{\text{dist}}=\sum_{x\in \Omega }{\left(\hat{D}(x)-D(x)\right)}^{2},$$

where *x* represents the pixel, Ω is the number of voxels in the whole brain. $\hat{D}(x)$ is the distance estimation map after activation of the Sigmoid function and $\hat{D}(x)$ is the ground truth distance map.

## Dataset and Experiments

### Datasets

**Figure 4:**
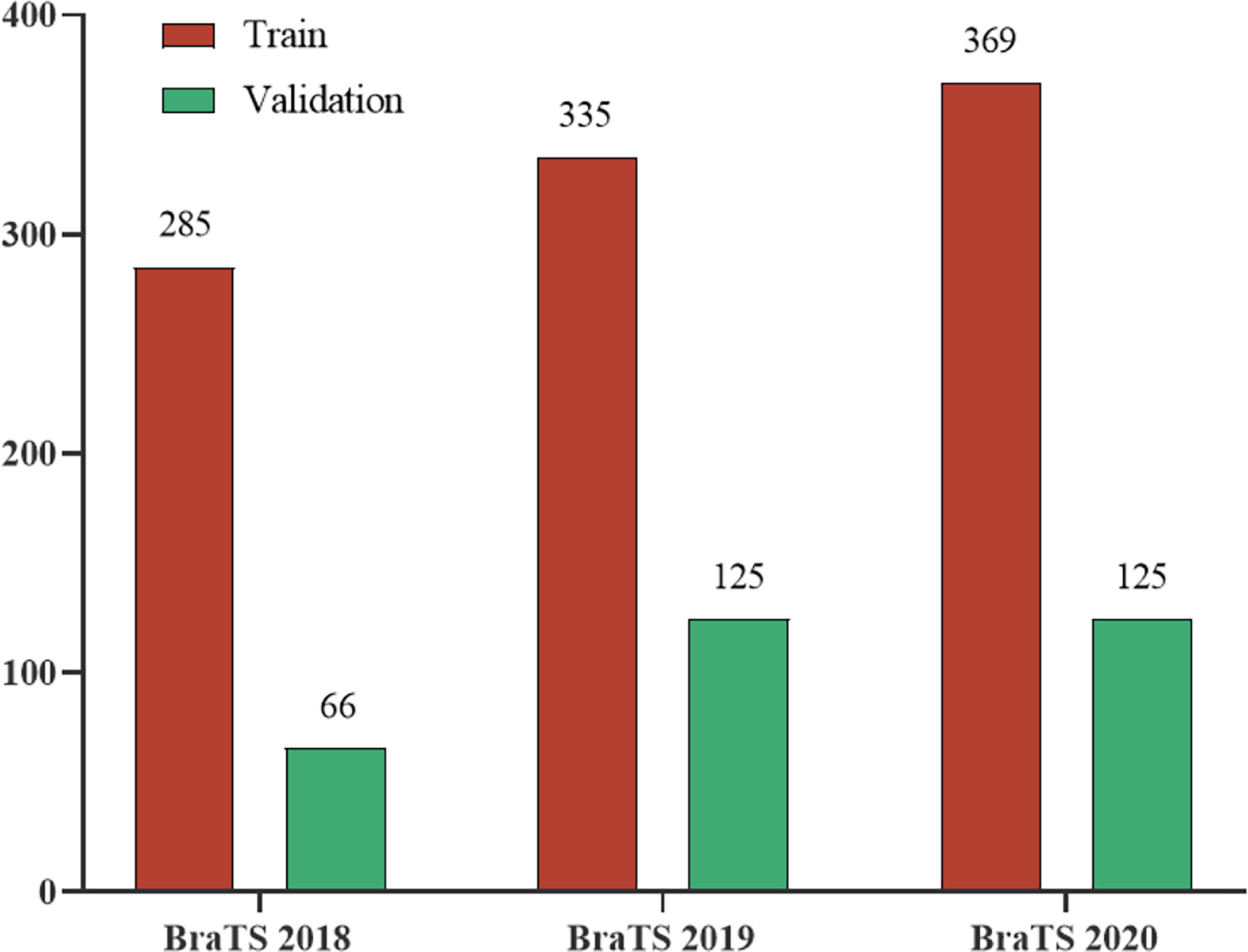
The division of the three datasets in the BraTS Challenge.

We evaluated our model on three different datasets, BraTS 2018, BraTS 2019, and BraTS 2020. These datasets all have two types of brain tumor data, namely high-grade glioblastoma (HGG) and low-grade glioma (LGG). The MRI of each sample contains four modes: fluid attenuation inversion recovery (FLAIR), T1 weighting (T1), T1-weighted contrast-enhanced (T1-CE), and T2 weighting (T2). The ground truth mask of the data has a necrotic area, edema area, and enhancement area. Our task is to segment the sub-regions formed by the nesting of three targets, the enhancing tumor (ET), the whole tumor (WT), and the tumor core (TC). They are all divided into the training set and unlabeled validation set by the organizer. Among them, BraTS2019 and BraTS2020 use the same test data, while the test data used by BraTS2018 is part of the 125 test data used by the former. The division of each dataset is shown in **Figure 4**.

### Evaluation Metrics

We follow the evaluation indicators of the brain tumor segmentation challenge in 2020, using Dice coefficient, sensitivity, specificity, and Hausdorff95 distance to measure the performance of the model. The Dice coefficient is an indicator of the overall evaluation, and its formula is defined as:

(6) $$\text{Dice}=\frac{2\text{TP}}{\text{FN}+\text{FP}+2\text{TP}},$$

where TP, FN, and FP represent the number of voxels of true positive, false negative, and false positive respectively. Sensitivity is used to measure the proportion of voxels in the tumor area that are correctly labeled, which can indicate the accuracy of the model segmentation of the target area, which is defined as:

(7) $$\text{Sensitivity}=\frac{\text{TP}}{\text{TP}+\text{FN}}.$$

Specificity represents the accuracy of the background voxel being correctly predicted, it can measure the ability of the model to predict the background area, defined as:

(8) $$\text{Specificity}=\frac{\text{TN}}{\text{TN}+\text{FP}},$$

where TN is the number of voxels with true negatives. Hausdorff95 distance measures the similarity between actual voxels and predicted voxels. The smaller the value, the closer the prediction is to the reality, it is defined as:

(9) $$\text{Haus}95(X,Y)=\text{max}\{\underset{x\in X}{\text{max}}\underset{y\in Y}{\text{min}d}(x,y),\underset{y\in Y}{\text{max}}\underset{x\in X}{\text{min}d}(x,y)\}$$

where *X* is the volume of ground truth, *Y* is the predicted volume, and d(.,.) is the distance from point x to point y.

### Post-Processing Steps

The most difficult part of BraTS dataset segmentation is to distinguish between enhanced tumors and tumor cores, especially when some tumor patients do not have enhanced tumors. If there is no enhanced tumor label in the ground truth and prediction, the BraTS Challenge will set the Dice score to 1. But if the ground truth does not enhance the tumor, even if there is only one false positive voxel in the prediction, the Dice score will be 0 (30). This greatly affects the Dice value in the ET area. Therefore, we post-process the segmentation results. If the total number of predicted enhanced tumors is less than 500, we replace all enhanced tumor voxels with tumor cores.

### Experimental Details

**Figure 5:**
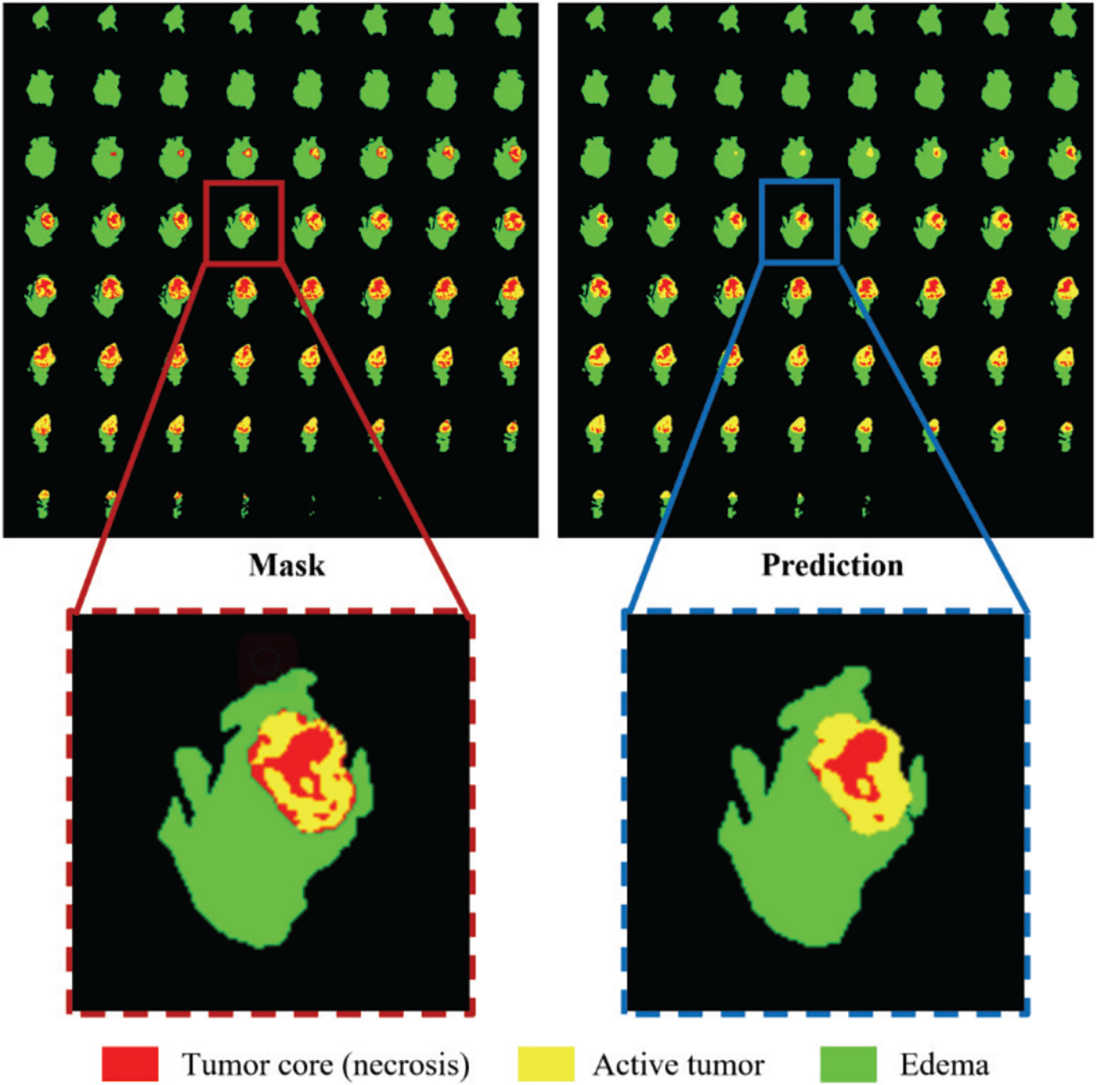
Comparison of the ground truth mask and the prediction during training. Red color represents the tumor core (necrosis), yellow color represents the active tumor and green regions are the edema.

All our implementations are based on Tensorflow 1.13.1, which is currently one of the most mainstream deep learning frameworks. Besides, we use the Adam optimizer to train the model. The specific details are that the entire network is trained for a total of 500,000 steps, and each training set is traversed about 10 times. After each traversal of the training set, the order of the data is randomly shuffled to enhance the robustness of training. The learning rate is initially set to 0.0001, and the training set is traversed twice, reducing to half of the original. Finally, we use Mean Dice as the evaluation index for our training and output the loss value and accuracy index every 10 steps to achieve effective supervision of model training. At the same time, the model outputs the segmentation effect map and the corresponding real label map every 1000 steps and saves the parameter model. In this way, the segmentation effect of the model is further monitored through the visualization method. The segmentation effect map and the real label map during the training process are shown in **Figure 5**.

TensorFlow platform is used for algorithm development using PyCharm with Python 3.6. The runtime platform processor is Intel (R) Xeon (R) Silver 4210 CPU @2.20GHz with 128GB RAM and Nvidia Titan RTX GPU on a 64-bit Windows 10 workstation. All algorithms are trained and tested using the same GPU and environment.

## Results and Discussion

### Results

The brain tumor segmentation method proposed in this paper is experimentally evaluated on three different datasets, namely BraTS 2018, BraTS 2019, and BraTS 2020. The preprocessing and segmentation process of these three datasets are the same. There are multi-modal imaging protocols in these datasets: Flair, T1, T1-CE, T2. The data comes from different centers, and the magnetic field strength is also different. The ground-truth was manually created by experts (31), including three nested sub-regions: the enhancing tumor (ET), the whole tumor (WT), and the tumor core (TC). In order to obtain better results, we train on 3D volumes. We use the Dice coefficient, sensitivity, specificity, and Hausdorff95 distance as the evaluation indicators of the model. **Tables 1** and **2** show the average results of the model on the validation sets, among them, “Post” represents the post-processing process added. The results show that the model achieves good segmentation results and has good robustness. Among them the segmentation accuracy of the WT and TC regions is high, but the segmentation accuracy of the ET region is slightly lower. The reason may be that the boundary between the ET region and the WT region is not obvious (32). We can see that post-processing has greatly improved the Dice accuracy in the ET area, with an average increase of 4%. The Dice value of all regions in the verification set of BraTS 2020 is greater than 0.75, especially the WT region is 0.86, which has exceeded the average level of existing methods. In particular, the specific value is stable at a high level, indicating that the model has stable performance in predicting the background area. At the same time, the sensitivity has also reached a very high level, and the accuracy of specificity is not much different, indicating that the model has a small difference in predicting target area and predicting background, and can effectively alleviate the problem of class imbalance in brain tumor segmentation. In MRI segmentation, multi-modal brain tumors are one of the most challenging tasks. Although our experimental results have some gaps compared with the top methods, we still achieve high accuracy.

**Table 1:** Comparison of Dice and Hausdorff95 post-processing of three validation sets.

	Dice			Hausdorff95		
	ET	WT	TC	ET	WT	TC
BraTS 2018	0.687	0.801	0.759	10.4	13.2	15.2
BraTS 2018+Post	0.717	0.801	0.759	9.9	13.2	15.2
BraTS 2019	0.700	0.827	0.788	6.6	8.5	9.2
BraTS 2019+Post	0.730	0.827	0.788	6.1	8.5	9.2
BraTS 2020	0.700	0.860	0.772	39.1	6.7	15.1
BraTS 2020+Post	0.750	0.860	0.772	34.6	6.7	15.1

**Table 2:** Comparison of Sensitivity and Specificity post-processing of three validation sets.

	Sensitivity			Specificity		
	ET	WT	TC	ET	WT	TC
BraTS 2018	0.789	0.962	0.800	0.996	0.977	0.995
BraTS 2018+Post	0.829	0.962	0.800	0.996	0.977	0.995
BraTS 2019	0.758	0.967	0.801	0.997	0.981	0.996
BraTS 2019+Post	0.798	0.967	0.801	0.997	0.981	0.996
BraTS 2020	0.749	0.958	0.791	0.999	0.998	0.999
BraTS 2020+Post	0.800	0.958	0.791	0.999	0.998	0.999

**Figure 6:**
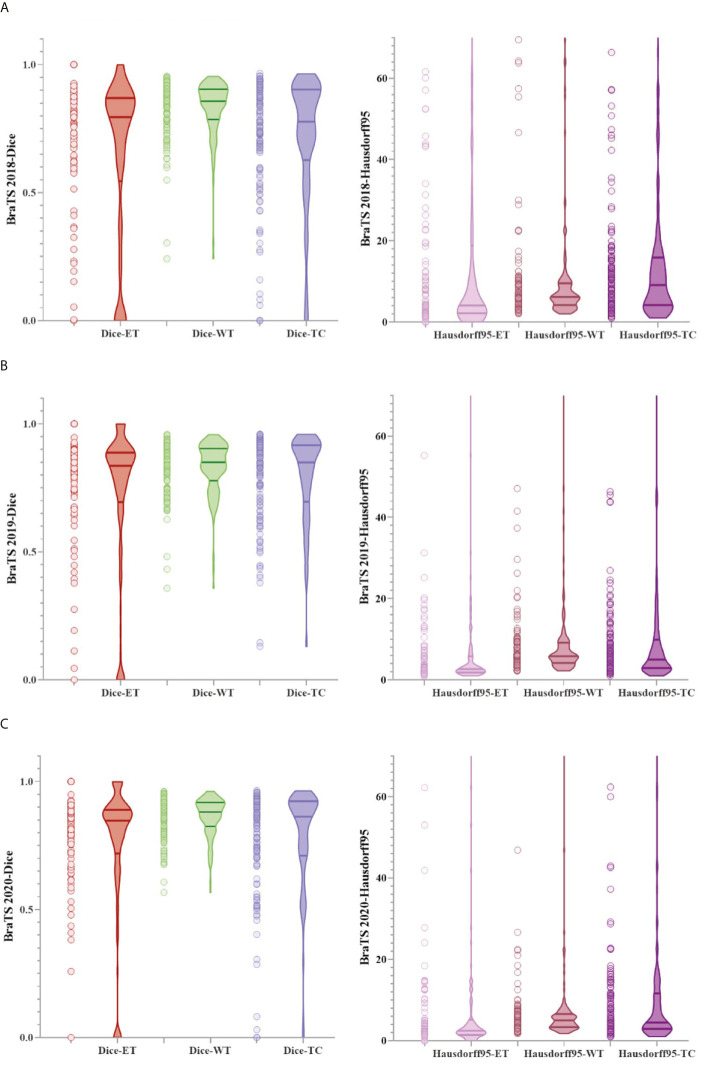
The result of the evaluation metrics of the validation set. **(A)** Dice and Hausdorff95 at BraTS 2018, **(B)** Dice and Hausdorff95 at BraTS 2019 and **(C)** Dice and Hausdorff95 at BraTS 2020.

**Figure 6** shows a combination of scatter plots and violin plots of the Dice and Hausdorff95 evaluation indicators in the three validation sets of BraTS. From the violin chart, it can be seen that the results are mainly concentrated in one area, and the median is obviously greater than the average, so the results are leftward and there are outliers. In the scatter plot, the data points are concentrated in areas with higher accuracy, which indicates that the model has a strong ability to predict individual situations.

**Figure 7:**
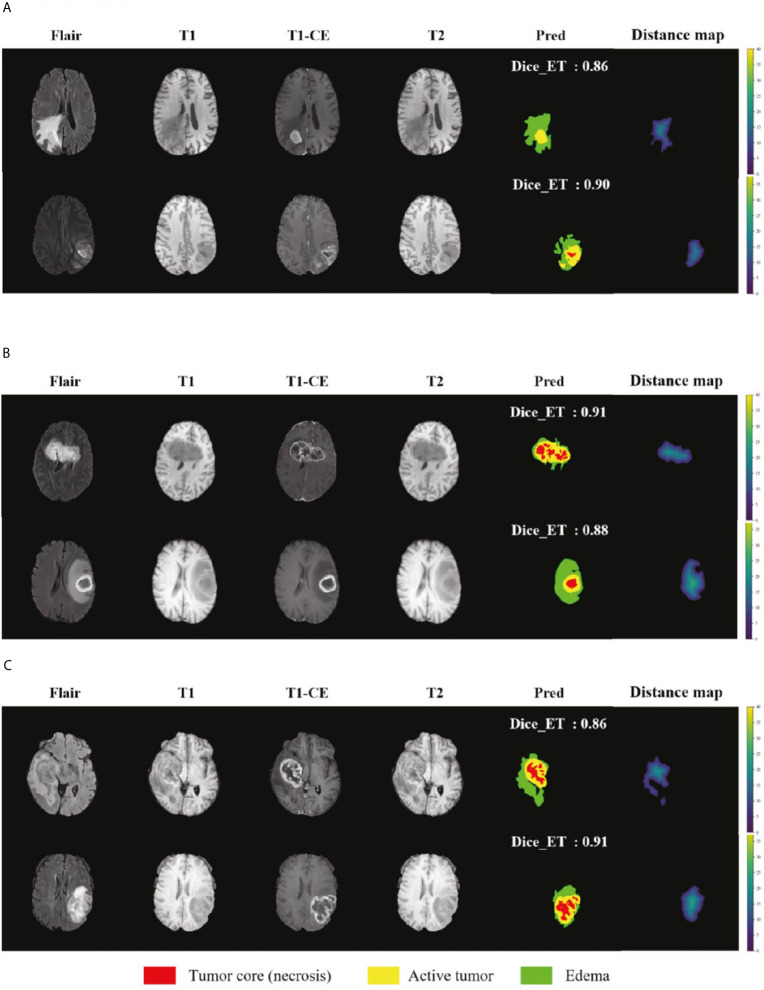
Display of the segmentation results in validation set samples. **(A)** BraTS 2018 Data, **(B)** BraTS 2019 Data and **(C)** BraTS 2020 Data. Red color represents the tumor core (necrosis), yellow color represents the active tumor and green regions are the edema.

We selected some representative segmentation results in the validation set for display, as shown in **Figure 7**. Among them (a) is the validation set of BraTS 2018, (b) is the validation set of BraTS 2019, and (c) is the validation set of BraTS 2020. For a more comprehensive display, we marked the Dice value of the ET area. **Figure 7** shows the segmentation results of the model in the validation set samples. From these examples, we can see that the model has good segmentation results for brain tumors of different sizes, positions, and shapes, and the predictive ability of the model is not affected by the intensity of MRI slice scans. Overall, the model has high performance.

### Comparison Study Results

In order to further verify the accuracy of the model, we compare the results of BraTS 2018 with other studies, as shown in **Table 3**, here only the values of Dice and Hausdorff95 are shown. Hu Y. et al. proposed a multi-level up-sampling network (MU-Net) to automatically segment multi-modal brain tumors. This model used the global attention GA module to combine the low-level feature maps obtained by the encoder with the high-level feature maps obtained by the decoder stand-up (33). Evan G. et al. used a 3-dimensional CNN constructed by the DeepMedic architecture created by Kamnitsas et al. (34), which contained a low-resolution and normal-resolution path, each with 11 layers (8). Tuan T. et al. proposed to segment all glioma regions using a U-Net model with multiple kernels (35). Weninger L. et al. first determined the location of the tumor and then used 3D U-Net to segment it (36). Hu X et al. proposed a 3D-Usid-Unet architecture, which included a context aggregation path and a localization path (37). Serrano-Rubio J.P. et al. trained an extreme random tree (ERT) algorithm to classify abnormal tissues with multiple labels (38).

**Table 3:** Comparison with the BraTS 2018 validation set of other methods.

BraTS 2018	Dice			Hausdorff95		
	ET	WT	TC	ET	WT	TC
**Proposed**	0.72	0.80	0.76	9.9	13.2	15.2
Evan G. et al	0.68	0.80	0.67	14.5	14.4	20.0
Hu Y. et al	0.66	0.87	0.72	7.56	6.73	15.74
Tuan T. et al	0.68	0.82	0.70	7.0	9.4	12.5
Weninger L. et al	0.71	0.86	0.82	5.6	7.0	7.9
Hu X. et al	0.72	0.86	0.77	5.5	10.8	10.0
Serrano-Rubio J. P. et al	0.60	0.84	0.73	11.7	9.0	14.7

We also compare the results of the BraTS 2019 dataset as shown in **Table 4**. Kim S. et al. used a two-step convolutional neural network (CNN) to segment brain tumors in brain MR images. First used three 2D U-Net to obtain the global information on the axial, coronal, and sagittal axes, and then used a 3D U-Net to obtain the local information in the 3D patch (39). The structure proposed by Amian M. et al. contained two parallel streamlines with two different resolutions. One was that the deep convolutional neural network learned the local features of the input data, and the other was to set the entire image for global observation. Then the output of each stream was combined to provide integrated learning of the input image (40). Shi W. et al. proposed a dense channel two-dimensional U-Net segmentation model based on residual units and feature pyramid units (41). Agravat R.R. et al. separately trained three tumor subcomponents and finally combined the three segmentation results to obtain a complete segmentation (42). Hamghalam M. et al. designed a novel pixel-by-pixel segmentation framework through a convolutional 3D to 2D MR patch conversion model (43). Wang F. et al. trained a deep learning model based on 3D U-net in the BraTS 2019 dataset with the help of brain intelligence and patching strategies (44).

**Table 4:** Comparison with the BraTS 2019 validation set of other methods.

BraTS 2019	Dice			Hausdorff95		
	ET	WT	TC	ET	WT	TC
**Proposed**	0.73	0.83	0.79	6.1	8.5	9.2
Kim S. et al	0.67	0.87	0.76	8.8	14.2	11.7
Amian M. et al	071	0.86	0.77	6.9	8.5	11.6
Shi W. et al	0.69	0.87	0.77	5.9	21.2	12.2
Agravat R.R. et al	0.60	0.70	0.63	11.69	14.33	17.10
Hamghalam M. et al	0.72	0.90	0.80	5.4	7.8	8.7
Wang F. et al	0.74	0.90	0.80	6.0	7.4	5.7

We compare the results with the teams participating in the 2020 BraTS Challenge to further demonstrate the effectiveness and generalization capability of our method. These data are available on the official website of the challenge as shown in **Table 5**. Based on the results of these comparisons, we analyze that most of the methods can only achieve a relatively high degree of accuracy in a certain sub-region. At present, there is no method to achieve the highest accuracy in all sub-regions. How to balance the accuracy of all segmented regions is also one of the directions of our future work.

**Table 5:** Comparison of validation set of teams participating in the BraTS 2020 challenge.

BraTS 2020	Dice			Sensitivity			Specificity			Hausdorff95		
	ET	WT	TC	ET	WT	TC	ET	WT	TC	ET	WT	TC
**Proposed**	0.75	0.86	0.77	0.80	0.96	0.79	0.99	0.99	0.99	34.6	6.7	15.1
DLU	0.70	0.87	0.78	0.72	0.89	0.78	0.99	0.99	0.99	39.8	8.9	11.2
MQUNSW	0.70	0.86	0.79	0.70	0.93	0.86	0.99	0.99	0.99	40.2	11.6	13.6
Nico@	0.67	0.87	0.71	0.62	0.84	0.65	0.99	0.99	0.99	41.7	10.1	33.5
agussa	0.59	0.83	0.69	0.60	0.87	0.71	0.99	0.99	0.99	56.6	23.2	30.0
FutureHealth	0.69	0.87	0.79	0.69	0.87	0.78	0.99	0.99	0.99	44.0	10.5	11.7
unet3d	0.70	0.84	0.72	0.71	0.87	0.79	0.99	0.99	0.99	37.4	12.3	13.1
Persistent	0.69	0.82	0.72	0.69	0.85	0.70	0.99	0.99	0.99	36.9	41.5	26.3
Iris	0.68	0.86	0.73	0.67	0.90	0.70	0.99	0.99	0.99	44.1	23.9	20.0
Uncertainty	0.68	0.87	0.78	0.66	0.90	0.77	0.99	0.99	0.99	47.6	12.1	15.7
ovgu_seg	0.60	0.79	0.68	0.66	0.78	0.67	0.99	0.99	0.99	54.1	12.1	19.1

### Discussions

We have shown that the proposed multi-task deep framework can be effectively used in multi-modal brain tumor segmentation tasks. At the same time, our integrated multi-depth fusion module can strengthen the feature extraction ability. The results on the 2020 BraTS dataset have shown the excellent performance of our model compared to other network structures. The Dice values of our ET, WT, and TC regions on the validation set are 0.75, 0.86, and 0.77, respectively, which are all above 0.7. The performance of our model is balanced among ET, WT, and TC and ranked #1, #2 and #3 compared to other state-of-the-art methods.

In order to show the effectiveness of the multi-depth fusion module in improving the segmentation accuracy, we train the multi-task model that does not include this module (Model I). **Table 6** shows its segmentation accuracy. Among them, the Dice scores in the ET, WT, and TC regions are 0.674, 0.848, and 0.747 respectively. Compared with the method proposed in this paper, it can be seen that the multi-depth fusion module can effectively improve the segmentation effect. Besides, when setting the weights of the loss function of the joint two tasks, we consider that the weight of the auxiliary task of distance estimation should not be greater than the weight of the segmentation task, so we set both *λ*_1_ and *λ*_2_ to 1. In our study, we have also tried different weight coefficients and set them to 1 and 0.1 for training (i.e., in Model II), and we have found that the accuracy of the segmentation has decreased. Therefore, we believe that the weights used in the article are locally optimal. The above comparison experiments are all completed on the BraTS 2020 dataset.

**Table 6:** Accuracy comparison with other comparative experiments.

BraTS 2020		Dice			Sensitivity			Specificity			Hausdorff95		
		ET	WT	TC	ET	WT	TC	ET	WT	TC	ET	WT	TC
Train	Proposed	**0.79**	**0.88**	**0.88**	**0.85**	0.97	**0.92**	**0.99**	**0.99**	**0.99**	28.4	**5.3**	**4.8**
	Model I	0.76	0.87	0.87	0.80	0.97	0.91	0.99	0.99	0.99	**23.1**	5.8	5.9
	Model II	0.73	0.85	0.87	0.82	**0.98**	0.91	0.99	0.99	0.99	28.8	7.0	6.6
Validation	Proposed	**0.75**	**0.86**	**0.77**	**0.80**	**0.96**	**0.79**	**0.99**	**0.99**	**0.99**	**34.6**	**6.7**	**15.1**
	Model I	0.67	0.85	0.75	0.72	0.94	0.76	0.99	0.99	0.99	42.1	7.4	18.8
	Model II	0.67	0.82	0.77	0.73	0.96	0.79	0.99	0.99	0.99	51.5	9.5	11.5

Bold values indicate the best performed method.

**Figure 8:**
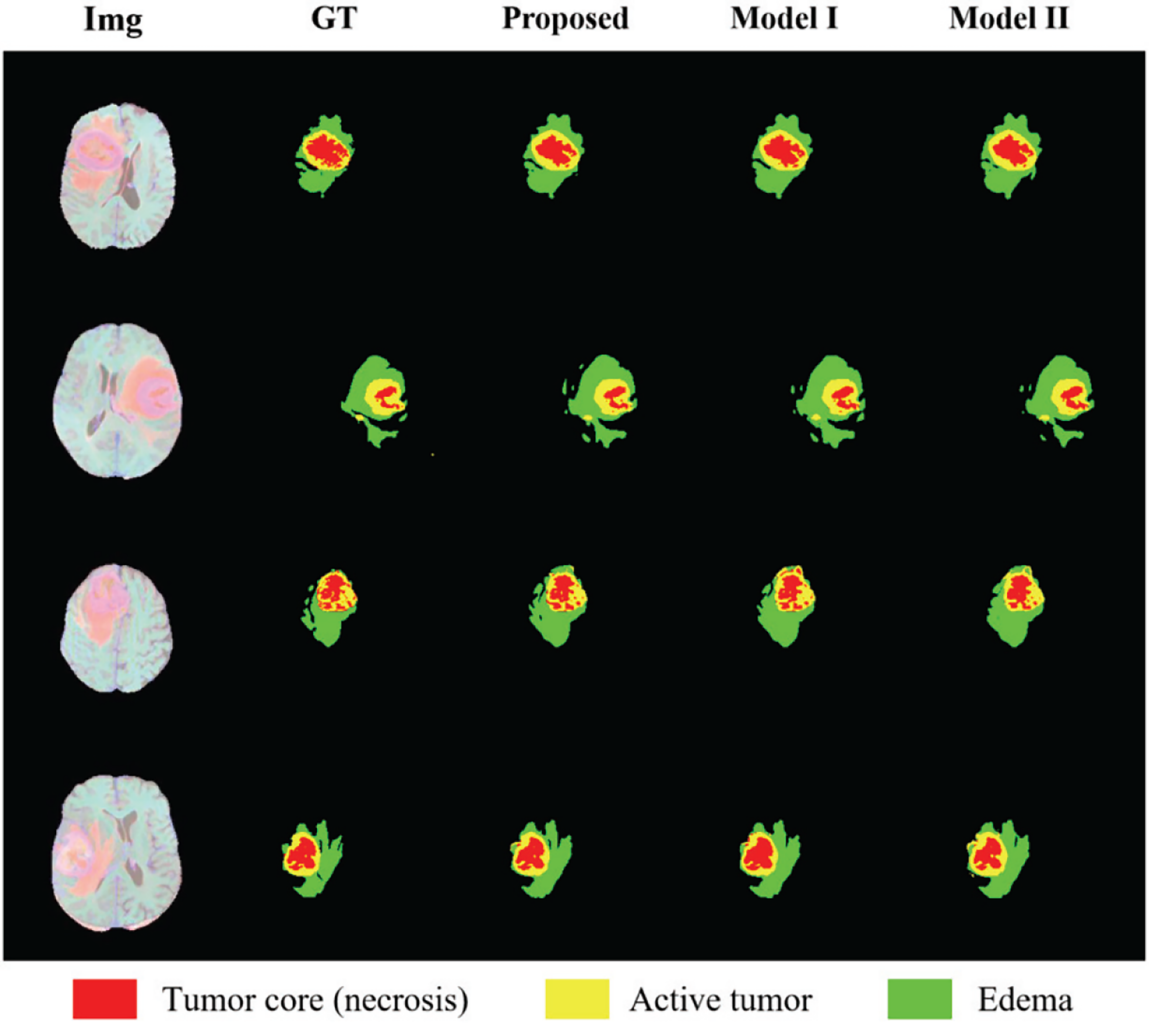
Results of randomly selected examples compared with other model variations. Red color represents the tumor core (necrosis), yellow color represents the active tumor and green regions are the edema.

In order to show the discrimination of the three methods more intuitively, we randomly select 4 examples in the training set for display as shown in **Figure 8**. Comparing the prediction results of the three models with the manually segmented labels, we can find that the model proposed in this paper has more advantages in detail prediction and has a more refined contour, which also proves the superiority of the model.

By comparing with some of the leading methods in this field, combined with some analysis of the results in this article, we have found that our method still has some limitations. First, in order to save training costs, we only set the weight coefficients of the loss function based on experience, and only did a set of experiments to adjust the weights instead of a large number of experiments to verify whether the weights are optimal to find the global optimal weights. In future research, we should find the optimal weight for many experiments to improve the accuracy of segmentation. Second, our segmentation results are not the best, and there are still some gaps compared with the top methods, so we propose the following improvement methods that can be developed in future work. Since the most clinically concerning information only occupies a small part of the image, inspired by Chen et al., we can design a method of region clipping, that is, to locate the part of interest (ROI) first and then segment it. This method can make the segmentation more precise. But this will also increase the amount of calculation. How to find a balance between accuracy and amount of calculation is also one of the focuses of our future work. Besides, the module we transplanted in the encoder proved to be suitable for whole-heart CT segmentation, but this is the first time that it is used for brain tumor segmentation, so we are not sure whether it is the most suitable attention module for brain tumor segmentation. We can try to use some other attention modules, such as the SE module, Non-local module, etc. After many comparison experiments, we can verify whether the effect of a multi-depth fusion module is the best.

## Conclusion

In this article, we propose a deep multi-task learning framework that integrates multi-depth fusion modules, and perform a performance test on multiple BraTS datasets, and obtain satisfactory results. We improved the traditional V-Net framework and proposed a structure of two parallel decoder branches. The original decoder can only perform segmentation, and the newly added decoder performs the auxiliary task of distance estimation, which can make the segmentation boundary more accurate. A total loss function is introduced to combine the two tasks. At the same time, we added a multi-depth fusion module after each encoder block to enhance the extraction of image features. We added a gamma factor to the loss function of the mask decoder to reduce the focus on the background area and set different weights for each type of label to alleviate the problem of category imbalance. We evaluated the accuracy of the model online for the BraTS 2018, BraTS 2019, and BraTS 2020 datasets. As a result, we obtained high-quality segmentation results, with an average Dice of 78%.

## Data Availability Statement

Publicly available datasets were analyzed in this study. This data can be found here: https://www.med.upenn.edu/sbia/brats2018/data.html.

## Ethics Statement

The studies involving human participants were reviewed and approved by Zhejiang Chinese Medical University. The ethics committee waived the requirement of written informed consent for participation.

## Author Contributions

HH performed the experiments, analyzed the data, and wrote the original draft of the manuscript. HH, GY, and XL performed the experiments, analyzed the data, and revised the manuscript. WZ and XX contributed to the experiments. WY and WJ contributed to the data analysis. GY and XL designed the project, supervised the experiments, drafted and revised the manuscript. All authors contributed to the article and approved the submitted version.

## Funding

This work is funded in part by the National Natural Science Foundation of China (Grant No. 62072413), and also supported in part by the AI for Health Imaging Award ‘CHAIMELEON: Accelerating the Lab to Market Transition of AI Tools for Cancer Management’ [H2020-SC1-FA-DTS-2019-1 952172].

## Conflict of Interest

The authors declare that the research was conducted in the absence of any commercial or financial relationships that could be construed as a potential conflict of interest.
